# *amdSYM*, a new dominant recyclable marker cassette for *Saccharomyces cerevisiae*

**DOI:** 10.1111/1567-1364.12024

**Published:** 2012-12-17

**Authors:** Daniel Solis-Escalante, Niels GA Kuijpers, Nadine Bongaerts, Irina Bolat, Lizanne Bosman, Jack T Pronk, Jean-Marc Daran, Pascale Daran-Lapujade

**Affiliations:** 1 Department of Biotechnology, Delft University of TechnologyDelft, The Netherlands; 2 Kluyver Centre for Genomics of Industrial FermentationDelft, The Netherlands; 3 Platform Green Synthetic BiologyDelft, The Netherlands

**Keywords:** counter-selectable marker, dominant marker, scarless marker removal, acetamidase, serial gene deletion, *Saccharomyces cerevisiae*

## Abstract

Despite the large collection of selectable marker genes available for *Saccharomyces cerevisiae*, marker availability can still present a hurdle when dozens of genetic manipulations are required. Recyclable markers, counterselectable cassettes that can be removed from the targeted genome after use, are therefore valuable assets in ambitious metabolic engineering programs. In the present work, the new recyclable dominant marker cassette *amdSYM,* formed by the *Ashbya gossypii TEF2* promoter and terminator and a codon-optimized acetamidase gene (*Aspergillus nidulans amdS*), is presented. The *amdSYM* cassette confers *S. cerevisiae* the ability to use acetamide as sole nitrogen source. Direct repeats flanking the *amdS* gene allow for its efficient recombinative excision. As previously demonstrated in filamentous fungi, loss of the *amdS* marker cassette from *S. cerevisiae* can be rapidly selected for by growth in the presence of fluoroacetamide. The *amdSYM* cassette can be used in different genetic backgrounds and represents the first counterselectable dominant marker gene cassette for use in *S. cerevisiae*. Furthermore, using astute cassette design, *amdSYM* excision can be performed without leaving a scar or heterologous sequences in the targeted genome. The present work therefore demonstrates that *amdSYM* is a useful addition to the genetic engineering toolbox for *Saccharomyces* laboratory, wild, and industrial strains.

## Introduction

The past decade has been marked by the construction of complex cell factories resulting from dozens of genetic manipulations and leading to remarkable new capabilities. For instance, the industrial and model yeast *Saccharomyces cerevisiae* has been engineered to produce the anti-malaria drug precursor artemisinic acid (Ro *et al*., 2006), hydrocortisone (Szczebara *et al*., 2003), and cineole (Ignea *et al*., 2011), among others. The ever-increasing demand for cheap and sustainable production of complex molecules combined with its attractiveness as a host for pathway engineering will inevitably intensify the exploitation of *S. cerevisiae* as cell factory in the future (Hong & Nielsen, 2012). Also, in fundamental yeast research, extensive genetic manipulation is necessary, for instance to unravel complex transport systems (Wieczorke *et al*., 1999; Suzuki *et al*., 2011) and regulatory networks (Baryshnikova *et al*., 2010). A common and critical feature for all these genetic manipulations is the requirement of selectable markers that enable the selection of mutants carrying the desired genetic modifications. Despite the relatively large number of selection markers available for *S. cerevisiae* (Table 1), the construction of multiple successive genetic modifications remains a challenge as the number of genetic manipulations typically equals the number of selection markers introduced in the host. Selection markers can be classified in two main categories: auxotrophic markers, which restore growth of specific mutants, and dominant markers, which confer completely new functions to their host. Both types suffer from substantial drawbacks. The use of auxotrophic markers is restricted to auxotrophic strains, that is, strains carrying mutations in one gene leading to a strict requirement for a specific nutrient (Pronk, 2002). This constrain is augmented for industrial strains that are typically prototrophic and for which the aneuploidy or polyploidy makes the construction of auxotrophic strains a laborious task (Puig *et al*., 1998). The expression in a single strain of multiple dominant marker genes, under the control of strong promoters, may result in protein burden and other negative effects on host strain physiology (Gopal *et al*., 1989; Nacken *et al*., 1996). Additionally, for industrial strains dedicated to food applications such as the production of nutraceuticals, the lack of heterologous DNA is highly desired.

**Table 1:** Different selectable markers used in laboratory and industrial *Saccharomyces cerevisiae* strains

Marker gene	Mode of action	Recyclable/Method	References
Auxotrophic markers			
*URA3*	Repairs uracil deficiency	Yes/negative selection with 5-FOA	Alani *et al*. (1987), Langlerouault & Jacobs (1995)
*KlURA3*	Repairs uracil deficiency	Yes/negative selection with 5-FOA	Shuster *et al*. (1987)
*CaURA3*	Repairs uracil deficiency	Yes/negative selection with 5-FOA	Losberger & Ernst (1989)
*HIS3*	Repairs histidine deficiency	No/–	Wach *et al*. (1997)
*HIS5*	Repairs histidine deficiency	No/–	Wach *et al*. (1997)
*LEU2*	Repairs leucine deficiency	No/–	Brachmann *et al*. (1998)
*KlLEU2*	Repairs leucine deficiency	No/–	Zhang *et al*. (1992)
*LYS2*	Repairs lysine deficiency	Yes/negative selection with alpha-aminoadipate	Chattoo *et al*. (1979)
*TRP1*	Repairs tryptophan deficiency	No/–	Brachmann *et al*. (1998)
*ADE1*	Repairs adenine deficiency	No/–	Nakayashiki *et al*. (2001)
*ADE2*	Repairs adenine deficiency	No/–	Brachmann *et al*. (1998)
*MET15*	Repairs methionine deficiency	Yes/negative selection with methyl-mercury	Singh & Sherman (1974), Brachmann *et al*. (1998)
Dominant markers			
*KanMX*	Resistance to G418	No/–	Wach *et al*. (1994)
*ble*	Resistance to phleomycin	No/–	Gatignol *et al*. (1987)
*Sh ble*	Resistance to Zeocin	No/–	Drocourt *et al*. (1990)
*hph*	Resistance to hygromycin	No/–	Gritz & Davies (1983)
*Cat*	Resistance to chloramphenicol	No/–	Hadfield *et al*. (1986)
*CUP1*	Resistance to Cu^2+^	No/–	Henderson *et al*. (1985)
*SFA1*	Resistance to formaldehyde	No/–	Van den Berg & Steensma (1997)
*dehH1*	Resistance to fluoroacetate	No/–	Van den Berg & Steensma (1997)
*PDR3-9*	Multi drug resistance	No/–	Lackova & Subik (1999)
*AUR1-C*	Resistance to aureobasidin	No/–	Hashida-Okado *et al*. (1998)
*nat*	Resistance to nourseothricin	No/–	Goldstein & McCusker (1999)
*CYH2*	Resistance to cycloheximide	No/–	Delpozo *et al*. (1991)
*pat*	Resistance to bialaphos	No/–	Goldstein & McCusker (1999)
*ARO4-OFP*	Resistance to o-Fluoro-DL-phenylalanine	No/–	Cebollero & Gonzalez (2004)
*SMR1*	Resistance to sulfometuron methyl	No/–	Xie & Jimenez (1996)
*FZF1-4*	Increased tolerance to sulfite	No/–	Cebollero & Gonzalez (2004)
*DsdA*	Resistance to d-Serine	No/–	Vorachek-Warren & McCusker (2004)

While protein burden might be avoided by expressing marker genes from inducible promoters (Suzuki *et al*., 2011), many inducible promoters are notoriously leaky (Agha-Mohammadi *et al*., 2004) and therefore only partly address the problem. A good alternative resides in the use of recyclable markers. Marker recycling was first shown with *URA3* (Alani *et al*., 1987). Loss of *URA3*, encoding orotidine-5′-phosphate decarboxylase involved in pyrimidine biosynthesis, is lethal in uracil-free media. Since its discovery, *URA3* has become a very popular auxotrophic selection marker. This popularity mainly originates from the ability of *URA3* to be counter-selected in the presence of 5-fluoroorotic acid (5-FOA), which is converted to a toxic compound (5-fluoro-UMP) by Ura3p. Indeed, when the *URA3* marker is flanked by direct repeats, cultivation on 5-FOA enables the efficient selection of strains in which *URA3* has been excised via mitotic recombination. *URA3* can therefore be re-used for further modifications (Alani *et al*., 1987; Langlerouault & Jacobs, 1995). Besides the obvious need for auxotrophic strains, another drawback of the most frequently used *URA3* cassette is that sequences (or scars) are left after marker removal. Commonly, the bacterial sequence *hisG* is present as direct repeat. However, repeated use of *URA3* cassettes carrying *hisG* increases the probability of mistargeted integrations (Davidson & Schiestl, 2000). An alternative to *hisG* is the creation of direct repeats upon integration using sequences already present in the host genome, generating seamless marker removal (Akada *et al*., 2006). Similar to *URA3*, two other auxotrophic markers *MET15* and *LYS2* can be counter-selected in the presence of methyl-mercury (Singh & Sherman, 1974) and alpha-aminoadipate (Chattoo *et al*., 1979), respectively. Nevertheless, their use is limited by the same complications described for *URA3*.

A successful attempt to recycle virtually any desired marker was the development of the bacteriophage-derived *Lox*P-*Cre* recombinase system (Hoess & Abremski, 1985; Sauer, 1987; Guldener *et al*., 1996, 2002). Exploiting the site-specific activity of the *Cre* recombinase, this system is used to efficiently remove markers by flanking them with the targeted *Lox*P sequence. This system exhibits two major limitations: (1) it requires the expression of a plasmid-borne recombinase and thereby necessitates an additional selection marker or extensive screening (Schorsch *et al*., 2009); and (2) the repeated use of this system causes major chromosomal rearrangements (Delneri *et al*., 2000; E. Boles, pers. commun.).

The *Aspergillus nidulans amdS* gene encoding acetamidase has been successfully used as dominant ‘gain of function’ selection marker in different filamentous fungi and the yeast *Kluyveromyces lactis* (Kelly & Hynes, 1985; Beri & Turner, 1987; Yamashiro *et al*., 1992; Swinkels *et al*., 1997; Selten *et al*., 2000; van Ooyen *et al*., 2006; Read *et al*., 2007; Ganatra *et al*., 2011). Although Selten *et al*. (2000) suggested that *amdS* could be used for selection in *S. cerevisiae*, this statement was not further supported by experimental evidence. Acetamidase catalyzes the hydrolysis of acetamide to acetate and ammonia, thus conferring the ability to the host cell to use acetamide as sole nitrogen or carbon source (Corrick *et al*., 1987; Hynes, 1994). Similar to *URA3*, *amdS* is a recyclable marker that can be counter-selected by growth on media containing the acetamide homologue fluoroacetamide, which is converted by acetamidase to the toxic compound fluoroacetate (Apirion, 1965; Hynes & Pateman, 1970).

This study evaluates the use of the new dominant marker module *amdSYM* for *S. cerevisiae* and demonstrates its efficiency for sequentially introducing multiple gene deletions in yeast. Availability of this dominant, counterselectable marker cassette to the yeast research community should facilitate rapid introduction of multiple genetic modifications into any laboratory, wild, and industrial *Saccharomyces* strains.

## Material and methods

### Strains and media

Propagation of plasmids was performed in chemically competent *Escherichia coli* DH5α according to manufacturer instructions (Z-competent™ transformation kit; Zymo Research, CA). All yeast strains used in this study are listed in Table 2. Under nonselective conditions, yeast was grown in complex medium (YPD) containing 10 g L^−1^ yeast extract, 20 g L^−1^ peptone, and 20 g L^−1^ glucose. Synthetic media (SM) containing 3 g L^−1^ KH_2_PO_4_, 0.5 g L^−1^ MgSO_4_·7H_2_O, 5 g L^−1^ (NH_4_)_2_SO_4_, 1 mL L^−1^ of a trace element solution as previously described (Verduyn *et al*., 1992), 1 mL L^−1^ of a vitamin solution (Verduyn *et al*., 1992) were used. When *amdSYM* was used as marker, (NH_4_)_2_SO_4_ was replaced by 0.6 g L^−1^ acetamide as nitrogen source and 6.6 g L^−1^ K_2_SO_4_ to compensate for sulfate (SM-Ac). Recycled markerless cells were selected on SM containing 2.3 g L^−1^ fluoroacetamide (SM-Fac). SM, SM-Ac, and SM-Fac were supplemented with 20 mg L^−1^ adenine and 15 mg L^−1^ l-canavanine sulfate when required. In all experiments, 20 g L^−1^ of glucose was used as carbon source. The pH in all the media was adjusted to 6.0 with KOH. Solid media were prepared by adding 2% agar to the media described above.

**Table 2:** Primers used for RT–qPCR, product sizes and efficiency values

Strain	Genotype	References
CEN.PK113-7D	*MAT*a *MAL2-8c SUC2*	van Dijken *et al*. (2000), Entian & Kotter (2007), Nijkamp *et al*. (2012)
CEN.PK113-5D	*MAT*a *MAL2-8c SUC2 ura3-52*	van Dijken *et al*. (2000), Entian & Kotter (2007)
CBS8066	*MATa/α HO/ho*	Centraal Bureau voor Schimmel-cultures, http://www.cbs.knaw.nl; CBS, Den Haag, the Netherlands
YSBN	*MATa/α ho::Ble/ho::HphMX4*	Canelas *et al*. (2010)
S288c	*MATα SUC2 gal2 mal mel flo1 flo8-1 hap1 ho bio1 bio6*	Mortimer & Johnston (1986)
CBS1483	*Saccharomyces pastorianu*s	Dunn & Sherlock (2008)
Scottish Ale	*Saccharomyces cerevisiae*	Gift from Dr. JM Geertman (Heineken Supply Chain, Zoeterwoude, the Netherlands)
CBS12357	*Saccharomyces eubayanus* sp.nov	Libkind *et al*. (2011)
IME141	*MAT*a *MAL2-8c SUC2 ura3-52* pAG426GPD *(2μ URA3 TDH3*_*pr*_*-CYC1*_*ter*_*)*	This study
IME142	*MAT*a *MAL2-8c SUC2 ura3-52* pUDE158 *(2μ URA3 TDH3*_*pr*_*-amdS-CYC1*_*ter*_*)*	This study
IMX168	*MAT*a *MAL2-8c SUC2 can1Δ::amdSYM ADE2*	This study
IMX200	*MAT*a *MAL2-8c SUC2 can1Δ ADE2*	This study
IMX201	*MAT*a *MAL2-8c SUC2 can1Δ ade2Δ::amdSYM*	This study
IMX206	*MAT*a *MAL2-8c SUC2 can1Δ ade2Δ*	This study
IMK468	*MAT*a *MAL2-8c SUC2 hxk1Δ::loxP-amdSYM-loxP*	This study
IMK470	CBS1483 *Sc-hxk1Δ::loxP-amdSYM-loxP*	This study
IMK473	Scottish Ale *Sc-aro80Δ::loxP-amdSYM-loxP*	This study
IMK474	CBS12357 *Seub-aro80Δ::loxP-amdSYM-loxP*	This study

### *amdSYM* and plasmid construction

A codon-optimized [JCat, http://www.jcat.de/, (Grote *et al*., 2005)] version of *A. nidulans amdS* flanked by SalI and XhoI restriction sites and *att*B1 and *att*B2 recombination sites (Accession number: JX500098) was synthesized at GeneArt AG (Regensburg, Germany) and cloned into the vector pMA (GeneArt AG) generating the plasmid pUD171. The *amdS* gene was transferred into the destination plasmid pAG426GPD (Alberti *et al*., 2007) by LR recombination reaction according to the manufacturer recommendations (Invitrogen, CA) yielding the plasmid pUDE158.

The *Ashbya gossypii TEF2*_ter_ from pUG6 (Guldener *et al*., 1996, 2002) was amplified, and the restriction sites XhoI and KpnI incorporated with the primers p1FW and p1RV and Phusion™ Hot Start Polymerase (Finnzymes, Vantaa, Finland). Restriction with XhoI and KpnI and ligation of the amplified fragment and the vector p426TEF (Mumberg *et al*., 1995) resulted in the replacement of the *CYC1*_ter_ of p426TEF for (*A.g*.) *TEF2*_ter_. The generated vector was termed p426TEF-TEF(t).

**Fig 1:**
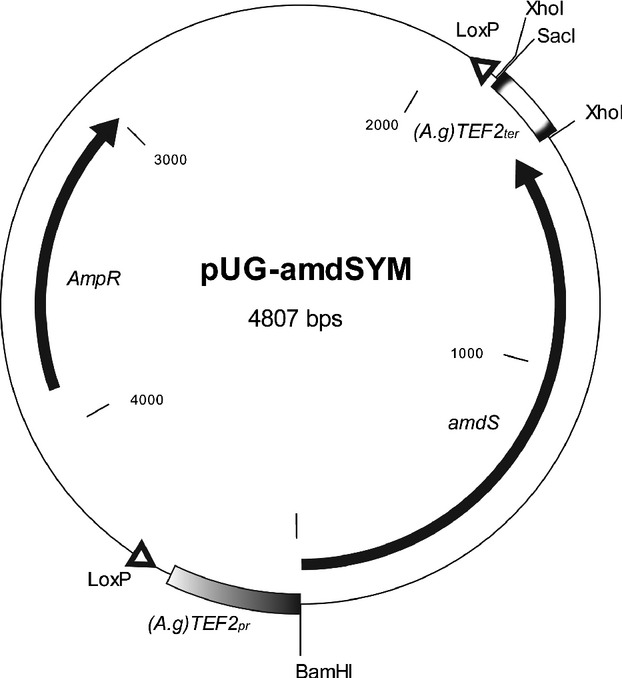
pUG-amdSYM map. pUG-amdSYM was constructed by replacing the marker *KanMX* in pUG6 for a codon-optimized version of *Aspergillus nidulans amdS*. The plasmid kept all features present in the pUG family (Guldener *et al*., 1996, 2002).

Primers amdSBFW and amdSXRV and Phusion™ Hot Start Polymerase (Finnzymes) were used to amplify *amdS* and to incorporate BamHI and XhoI sites using pUD171 as template. After digestion with the corresponding enzymes, the gene was cloned into the vector p426TEF-TEF(t) generating the plasmid pUD184. Using primers amdSBFW and amdSSRV, containing BamHI and SacI restriction sites, respectively, and Phusion™ Hot Start Polymerase (Finnzymes), the gene *amdS* together with *A. gossypii TEF2* terminator was amplified from pUD184. To incorporate BamHI and SacI restriction sites in pUG6, the complete plasmid backbone was amplified by PCR with the primers SpUGFW and pUGBRV and Phusion™ Hot Start Polymerase (Finnzymes). Digestion with BamHI and SacI of the modified pUG6 and the *amdS-(A.g).TEF2*_*ter*_ and ligation led to the construction of pUG-amdSYM (Fig. 1). All primers and their sequences are listed in Table 3. The plasmid pUG-amdSYM is available to the research community at Euroscarf (http://web.uni-frankfurt.de/fb15/mikro/euroscarf/) with the accession number: P30669.

**Table 3:** Primers used in this study

Primer	Sequence 5′–3′*^,^†
Plasmid construction	
amdSBFW	GGGGGATCCATGCCACAATCTTGGGAAGAA
amdSXRV	GGGCTCGAGTTATGGAGTAACAACG
amdSSRV	GGGGAGCTCCAGTATAGCGACCAGCATTC
p1FW	CCGCTCGAGCAGTACTGACAATAAAAAGATTC
p1RV	GCGGTACCCAGTATAGCGACCAGCATTC
pUGBRV	GGGGGATCCTTGTTTATGTTCGGATGTGATGTG
SpUGFW	GGGCGAGCTCTCGAGAACCCTTAA
Deletion cassette construction	
CdcamdSFW	TCAGACTTCTTAACTCCTGTAAAAACAAAAAAAAAAAAAGGCATAGCAATCAGCTGAAGCTTCGTACGC
CdcamdSRV	ATGCGAAATGGCGTGGGAATGTGATTAAAGGTAATAAAACGTCATATCTA**AGAACTCTGAAATAAACTTTCGATTGACGACAGATTGAAA**GCATAGGCCACTAGTGGATCTG
AdcamdSFW	TACTATAACAATCAAGAAAAACAAGAAAATCGGACAAAACAATCAAGTATCAGCTGAAGCTTCGTACGC
AdcamdSRV	TCATTTTATAATTATTTGCTGTACAAGTATATCAATAAACTTATATATTA**GGATGTACTTAGAAGAGAGATCCAACGATTTTACGCACCA**GCATAGGCCACTAGTGGATCTG
HdcamdSFW	AAACTCACCCAAACAACTCAATTAGAATACTGAAAAAATAAGATGATGACAAGAGGGTCGAACTCCAGCTGAAGCTTCGTACGC
HdcamdSRV	AGGGAGGGAAAAACACATTTATATTTCATTACATTTTTTTCATTAGCCTAAGTCGTAATTGAGTCGCATAGGCCACTAGTGGATCTG
ScHdcamdSFW	CTTCTTATGCCCCTGAACCC
ScHdcamdSRV	CTATCCTACGACTTTCTCCCTC
*ScARO80*dcamdSFW	AGTTAGTCGTAGGAATATATGATCCACGCATAATAAGGTTACATTAAGCACTGCTTTATCCAGCTGAAGCTTCGTACGC
*ScARO80*dcamdSRV	CTTTGTATTTAAAATCATTTTTACGAATAGTGCGGTTGTCTTGGTTGATGACGTAATTCTGCATAGGCCACTAGTGGATCTG
*ScARO80*g5′FW	CACTACCAAAGCCAAATCAGAC
*ScARO80*g5′RV	GATAAAGCAGTGCTTAATGTAACC
*ScARO80*g3′FW	AGAATTACGTCATCAACCAAGAC
*ScARO80*g3′RV	AGCGTAGCTTGCACTACTAG
*SeubARO80*dcamdSFW	AGCAAGCTAGTCAAAATATTTGCTCTCCGCATGATATAATTACTTTCAGTATCGTTGTCCCCAGCTGAAGCTTCGTACGC
*SeubARO80*dcamdSRV	TAGTAATCAATCATTTATCTAATTAAACATTCCTTTTCTCTAATTTTATGTGTGAGGGGCGCATAGGCCACTAGTGGATCTG
*SeubARO80*g5′FW	GACATTGATGACAATGGTAGTG
*SeubARO80*g5′RV	GGACAACGATACTGAAAGTAAT
*SeubARO80*g3′FW	GCCCCTCACACATAAAATTAGAG
*SeubARO80*g3′RV	GTGTGGCTTGTACCACAAGA
Deletion/Marker removal confirmation	
CdcFW	CGGGAGCAAGATTGTTGTG
CdcRV	GGTTGCGAACAGAGTAAACC
AdcFW	AAAGGACACCTGTAAGCGTTG
AdcRV	AGCATTTCATGTATAAATTGGTGCG
HdcFW	CCTTAGGACCGTTGAGAGGAATAG
HdcRV	GACCGCAAAAAAAACATAAGGG
*ScARO80*dcFW	TGATCCCGATACTGGAAATCAA
*amdS*dcRV	CGACCAGCATTCACATACGA
*ScARO80*gRV	TTCATCCTATCTGAACAGAATAC
*SbARO80*dcFW	GTCATGCAGGCTCTTCATTG
*SbARO80*gRV	TATCCCTCACGTGAATTTAAACC
Sequencing	
C-FW	ATCACTTACTGGCAAGTGCG
C-RV	ATCAGTTGTGCCTGGAAAAG
A-FW	AACGCCGTATCGTGATTAAC
A-RV	GGACACTTATATGTCGAGCAAGA

* The sequences underlined indicate the position of the introduced restriction.

† †The sequences in bold indicate the direct repeats used to excise the marker gene upon counter selection.

### Deletion cassette construction

Deletion cassettes were constructed by PCR using Phusion™ Hot Start Polymerase (Finnzymes) and following manufacturer recommendations. Primers used for repeated gene deletions had a similar design described as follows. Forward primers contain two cores: (1) a 50- to 55-bp sequence homologous to the region upstream the gene to delete and (2) the sequence 5′-CAGCTGAAGCTTCGTACGC-3′ that binds to the region upstream the *A. gossypii TEF2* promoter in pUG-amdSYM. Reverse primers contained three cores: (1) a 50- to 55-bp sequence homologous to the region downstream the gene to be deleted, (2) a 40-bp sequence homologous to the region upstream the targeted region, and (3) the sequence 5′-GCATAGGCCACTAGTGGATCTG-3′ that binds downstream the *A. gossypii TEF2* terminator in pUG-amdSYM. *CAN1* and *ADE2* deletion cassettes were constructed using the primer pairs CdcamdSFW and CdcamdSRV, and AdcamdSFW and AdcamdSRV, respectively (Table 3).

To construct the deletion cassette targeting *S. cerevisiae HXK1* (*Sc.HXK1*), primers HdcamdSFW and HdcamdSRV and the template pUG-amdSYM were used. The constructed cassette was used to generate the strain IMK468 derived from CEN.PK113-7D. Genomic DNA of IMK468 was used as template for primers ScHdcamdSFW and ScHdcamdSRV. The resulting cassette contained 500-bp homologous sequences upstream and downstream of the *Sc.HXK1* gene and was used for the deletion of *Sc.HXK1* in the *Saccharomyces pastorianus* lager brewing strain CBS1483. The construction of the deletion cassette corresponding to *Sc.ARO80* allele for the *S. cerevisiae* Scottish Ale strain was performed according to the two-step fusion protocol (Amberg *et al*., 1995) with the primers ScARO80dcamdSFW and ScARO80dcamdSRV to amplify *amdSYM* from pUG-amdSYM, and ScARO80g5′FW, ScARO80g5′RV, ScARO80g3′FW, and ScARO80g3′RV to generate the 500-bp sequence homologous to upstream and downstream sections of *Sc.ARO80*, and genomic DNA from CBS1483 was used as template. The same approach was taken for the deletion of *Saccharomyces eubayanus ARO80* (*Sb.ARO80*) in CBS12357 using the primer pairs SeubARO80dcamdSFW/SeubARO80dcamdSRV, SeubARO80g5′FW/SeubARO80g5′RV, and SeubARO80g3′FW/SeubARO80g3′RV. All primers and their sequences are listed in Table 3.

### Selection and marker recycling

Yeast transformations were performed using the lithium acetate protocol (Gietz & Woods, 2002). Integration of the deletion cassettes into the yeast genome was selected by plating the transformation mix on SM-Ac. Targeted integration was verified by PCR with the primer pairs CdcFW/CdcRV, AdcFW/AcdRV, HdcFW/HdcRV, ScARO80dcFW/amdSdcRV, ScARO80dcFW/ScARO80gRV, SbARO80dcFW/amdSdcRV, SbARO80dcFW/SbARO80gRV and, when applicable, by transferring single colonies to SM plates containing 15 mg L^−1^ l-canavanine or by screening for colony pigmentation. A small fraction of single colony was resuspended in 15 μL of 0.02 N NaOH; 2 μL of this cell suspension was used as template for the PCR that was performed using DreamTaq PCR master mix (Fermentas GmbH, St. Leon-Rot, Germany) following the manufacturer recommendations. Marker removal was achieved by growing cells overnight in liquid YPD and transferring 0.2 mL to a shake flask containing 100 mL of SM-FAc. Marker-free single colonies were obtained by plating 0.1 mL of culture on SM-Fac solid media and confirmed by PCR with the primer pairs CdcFW/CdcRV or AdcFW/AcdFW. All cultures were incubated at 30 °C. To confirm that only endogenous sequences were present after marker removal, long run sequencing (Baseclear, Leiden, the Netherlands) was performed using the primers C-FW and C-RV for the *CAN1* locus and A-FW and A-RV for *ADE2* locus of the marker-free strain IMX206.

## Results and discussion

### Expression of *amdS* in *S. cerevisiae* confers the ability to grow on acetamide as sole nitrogen source

Although the yeast putative amidase gene *AMD2* is similar (57.2% similarity and 33.2% identity) to *A. nidulans amdS* (Chang & Abelson, 1990), there is no report demonstrating acetamidase activity in wild-type *S. cerevisiae* or on growth of budding yeast on acetamide as sole nitrogen or carbon source. Expression of *amdS* in *S. cerevisiae* is therefore expected to bring a new function in this yeast by enabling its growth on acetamide as sole nitrogen source. Although the *Aspergillus nidulans amdS* promoter is able to drive the expression of genes in *S. cerevisiae*, this is only possible under carbon-limited conditions (Bonnefoy *et al*., 1995). Therefore, a codon-optimized *amdS* sequence was cloned under the control of the strong, constitutive *TDH3* promoter in the plasmid pUDE158.

**Fig 2:**
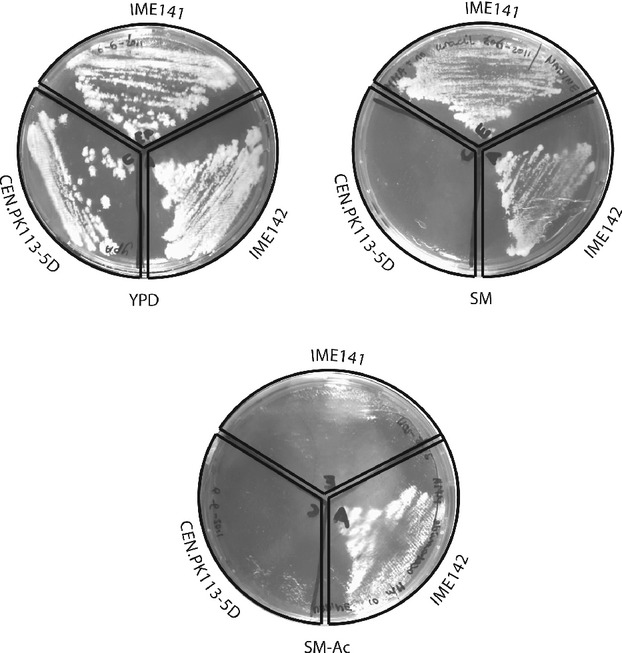
Growth of strains from the CEN.PK family on acetamide as sole nitrogen source. Expression of *amdS* on the multicopy plasmid pUDE158 conferred to *Saccharomyces cerevisiae* the ability to grow on acetamide as sole nitrogen source. The strains CEN.PK113-5D (*ura3-52*), IME141 [*ura3-52* pAG426GPD (*2μ URA3 TDH3*_*pr*_*-CYC1*_*ter*_)], and IME142 [*ura3-52* pUDE158 (*2μ URA3 TDH3*_*pr*_*-amdS-CYC1*_*ter*_)] were grown on YPD, SM and SM-Ac media and incubated at 30 °C. The plates were read after 3 days.

The plasmid pUDE158, containing *amdS*, and the empty vector pAG426GPD were transformed into CEN.PK113-5D, generating strains IME142 and IME141, respectively. Expression of the acetamidase gene in *S. cerevisiae* (strain IME142) conferred growth with acetamide as sole nitrogen source, while the control strain (strain IME141) was unable to grow (Fig. 2). Additionally, the inability of IME142 to grow on acetamide upon loss of *amdS* by counter selection with 5-FOA of pUDE158 confirmed that growth on acetamide was fully *amdS* dependent (data not shown).

### Plasmids and deletion cassettes construction

The coding sequence of the *A. nidulans amdS* gene, codon-optimized for expression in *S. cerevisiae* and flanked by the *A. gossypii TEF2* promoter and terminator, was cloned into the vector pUG6 (Guldener *et al*., 1996, 2002) by replacing the *KanMX* gene, resulting in the plasmid pUG-amdSYM (Fig. 1). The resulting *amdSYM* module only contained heterologous sequences, thereby reducing the probability of mistargeted integration (Wach *et al*., 1994). The pUG-amdSYM plasmid can be easily used as template for deletion cassettes containing the new marker module *amdSYM* and was used for the construction of all deletion cassettes used in this study.

**Fig 3:**
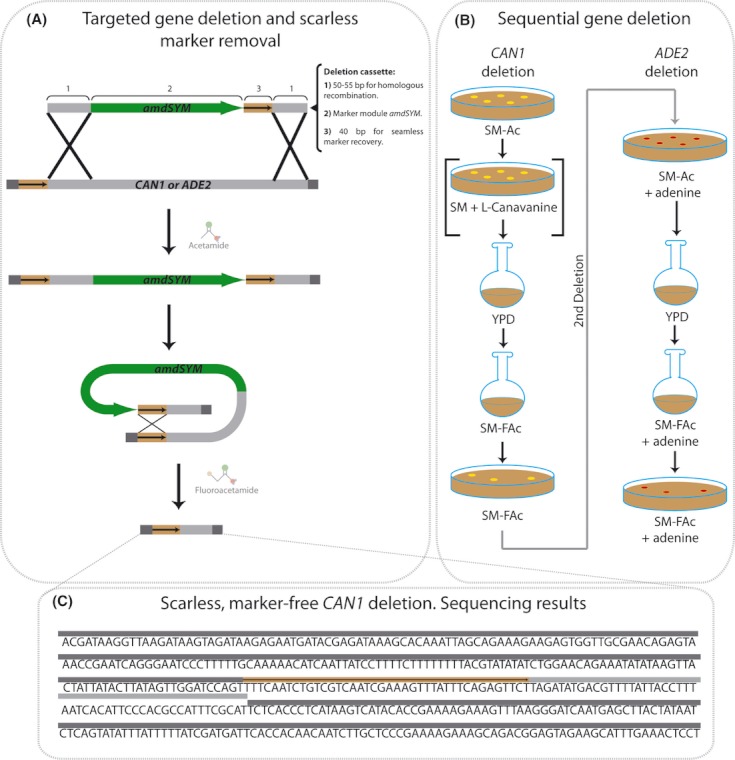
Sequential gene deletions methodology. (A) Cassette design for targeted gene deletion and seamless marker removal. (B) Experimental procedure for the sequential deletion of *CAN1* and *ADE2* in *Saccharomyces cerevisiae* by *amdSYM* recycling. (C) Sequencing results of the *CAN1* loci of the marker-free IMX206 (*can1Δ ade2Δ*).

The deletion cassettes contained three major regions (Fig. 3A): (1) a 50- to 55-bp sequence homologous to the upstream part of the gene to be deleted, including the start codon, and a 50- to 55-bp sequence homologous to the downstream part of the gene to be deleted, including the stop codon. These regions were used for targeted homologous recombination (Baudin *et al*., 1993): (2) the *amdSYM* marker and (3) a 40-bp sequence homologous to the region upstream of the targeted locus to create direct repeats in the host genome upon integration, thereby enabling scarless marker excision (Akada *et al*., 2006; Fig. 3).

### Repeated gene deletions in *S. cerevisiae* using *amdSYM*

To evaluate whether the new marker *amdSYM* was suitable for repeated gene knock-out in *S. cerevisiae*, it was attempted to sequentially delete two genes in the laboratory strain CEN.PK113-7D and, after marker recycling, to construct a marker-free and scarless double-deletion strain. *CAN1* and *ADE2* were selected for this proof-of-principle experiment because the phenotype caused by *CAN1* or *ADE2* deletion can be visually screened, giving a fast preliminary evaluation of targeted integration. *CAN1* encodes an l-arginine transporter that can also import the toxic compound l-canavanine. Mutants with disrupted *CAN1* are able to grow in media containing l-canavanine (Ahmad & Bussey, 1986). *ADE2* codes for the enzyme phosphoribosylaminoimidazol carboxylase, which is involved in the biosynthesis of purine nucleotides. *ade2* mutants require an external source of adenine and accumulate precursors of purine nucleotides in the vacuole which give colonies a red color (Zonneveld & Vanderzanden, 1995; Fig. 3B).

**Fig 4:**
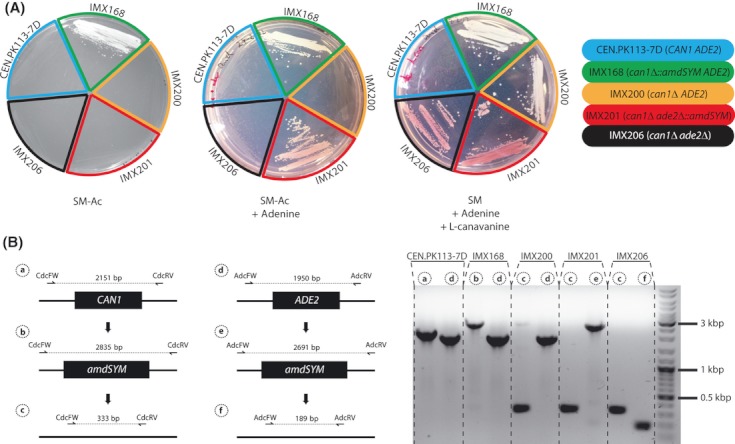
Sequential gene deletions of *CAN1* and *ADE2* using *amdSYM* in *S. cerevisiae*. (A) The strains IMX168, IMX200, and IMX201 and the parental strain CEN.PK113-7D were grown on SM-Ac, SM-Ac supplemented with adenine, and SM supplemented with adenine and l-canavanine. The plates were incubated at 30 °C and were read after 3 days. (B) PCR analysis to confirm correct integration of the gene disruption cassettes and their removal at the *CAN1* and *ADE2* loci. PCR was carried out on reference CEN.PK113-7D, IMX168, IMX200, and IMX201. All PCRs were performed with the primer pairs CdcFW/CdcRV and AdcFW/AdcRV for *CAN1* and *ADE2* loci, respectively. In the parental strain, amplification of the *CAN1* and *ADE2* loci generated fragments of 2151 bp (a) and 1950 bp (d) for *CAN1* and *ADE2*, respectively. PCR on IMX168 DNA generated a fragment of 2835 bp (b) due to the incorporation of *amdSYM* in the *CAN1* locus. A short fragment of 333 bp (c) was obtained for IMX200 as a result of *amdSYM* excision from the *CAN1* locus. Similarly, the disruption of *ADE2* using *amdSYM* led to a large PCR product of 2691 bp in IMX201 (e) while PCR on the *ADE2* locus in the marker-free strain IMX206 generated a short fragment of 189 bp (f). The products obtained were then subjected to agarose gel electrophoresis.

The potential of *amdSYM* as dominant marker was tested by transforming a deletion cassette to disrupt *CAN1* in CEN.PK113-7D. After transformation, cells were grown on synthetic medium (SM) agar plates containing acetamide as sole nitrogen source (SM-Ac, Fig. 4A). Targeted gene deletion was confirmed by the ability of single colonies to grow on SM containing l-canavanine (Fig. 4A) and by PCR (Fig. 4B). The average transformation efficiency was 14 transformants μg^−1^ of DNA, with 100% of the colonies harboring the correct integration. It is important to note that *amdSYM* did not yield false positives. Although the transformation efficiency reported here for the deletion of *CAN1* may appear low as compared to efficiencies reported for other dominant markers such as *KanMX* (100 transformants μg^−1^ DNA) (Guldener *et al*., 1996), in our experience, the targeted region and the sequence of the deletion cassette affects much more the transformation efficiency than the nature of the marker used. To support this, it has recently been shown that nucleosome density is a critical factor for transformation efficiency, thereby demonstrating that the localization of a deletion cassette has a strong impact on the transformation efficiency (Aslankoohi *et al*., 2012). With deletions cassettes using *amdSYM*-based selection but targeted to other loci than *CAN1*, we observed transformation efficiencies similar to those reported for classical selection markers (data not shown).

The resulting strain IMX168 was subsequently used to evaluate the potential of marker excision aided by the direct repeats created upon integration (Fig. 3B). Under nonselective conditions, that is, growth in complex media (YPD), mitotic recombination between the direct repeats flanking *amdSYM* may excise the marker. To select for these recombinants, strain IMX168 (*can1Δ::amdS*) was grown overnight in liquid complex media, transferred to synthetic medium containing fluoroacetamide (SM-Fac), and then plated on SM-Fac. PCR analysis of the growing colonies confirmed correct marker removal (Fig. 4B); the new strain was named IMX200 (*can1Δ*). Due to the absence of *CAN1*, IMX200 (*can1Δ*) was able to grow on media containing l-canavanine. As anticipated, it had lost the ability to grow on media containing acetamide as nitrogen source due to the removal of *amdSYM* (Fig. 4A). These results confirmed that *amdSYM* can be successfully used as a selection marker in the prototrophic laboratory strain CEN.PK113-7D and that the marker can be removed to avoid protein burden. An important addition is that the design of the deletion cassette allows for marker removal leaving only endogenous sequences (Fig. 3C).

A strong feature of recyclable markers is that a single marker is sufficient to perform multiple sequential manipulations in the same strain. The potential of *amdSYM* for serial deletion was tested by deleting a second gene in the marker-free strain IMX200 (*can1Δ*). After transformation with an *amdSYM* deletion cassette targeted to *ADE2* locus, correct transformants could be easily selected for their ability to grow in the presence of l-canavanine and to use acetamide as nitrogen source and for their auxotrophy for adenine and red pigmentation (Fig. 4A). The strain IMX201 (*can1Δ ade2Δ::amdS*) was selected using these criteria, and correct integration of the deletion cassette was confirmed by PCR (Fig. 4B). This second deletion demonstrated that *amdSYM* is a powerful selection marker for serial gene deletion. IMX201 was further engineered to generate the marker-free strain IMX206 (*can1Δ ade2Δ*) by scarless removal of *amdSYM*. This was confirmed by sequencing the *CAN1* and *ADE2* loci in IMX206. Similar to the parental strain CEN.PK113-7D, strain IMX206 (*can1Δ ade2Δ*) was unable to grow on media containing acetamide as sole nitrogen source but, due to the deletions performed, showed a red pigmentation, was not able to grow in absence of adenine, and was able to grow on media containing l-canavanine (Fig. 4). This marker- and scar-free strain can be subsequently used for additional deletions or other genetic manipulations.

### The module *amdSYM* can be used in a wide range of laboratory, wild, and industrial *Saccharomyces* strains

**Fig 5:**
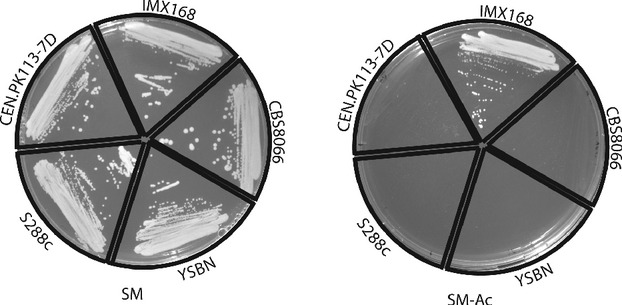
Growth of *Saccharomyces cerevisiae* laboratory strains on acetamide as sole nitrogen source. The laboratory strains CEN.PK113-7D, CBS8066, YSBN, S288c, and the modified strain IMX168 (*can1Δ::amdSYM*) were grown on SM-Ac and SM media and incubated at 30 °C. The plates were read after 3 days. Only the strain harboring the *amdSYM* module, IMX168, was able to grow when acetamide was used as nitrogen source.

**Fig 6:**
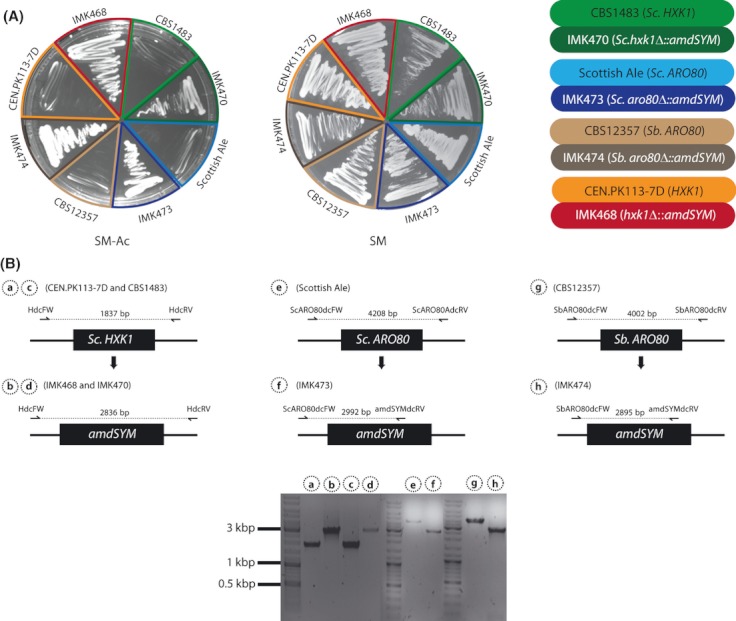
*amdSYM* as selectable marker for laboratory, wild, and industrial *Saccharomyces* strains. (A) The laboratory strain CEN.PK113-7D, the industrial strains CBS1483, Scottish Ale and the wild *Saccharomyces eubayanus* CBS12357, and the generated strains IMK468, IMK470, IMK473, and IMK474 were grown on SM-Ac and SM media and incubated at 30 °C. The plates were read after 5 days. (B) PCR analysis to confirm correct integration of the gene disruption cassettes was carried out on CEN.PK113-7D, CBS1483, Scottish Ale, CBS12357, IMK468, IMK470, IMK473, and IMK474. Amplification of *Sc*.*HXK1* locus in CEN.PK113-7D and CBS1483 generated fragments of 1837 bp (a, c), bigger fragments, 2836 bp (b, d) were obtained in the strains IMK468 and IMK470 due to the integration of *amdSYM*. Amplification of the loci *Sc*.*ARO80* in Scottich Ale and *Sb*.*ARO80* in CBS12357 generated fragments of 4208 bp (e) and 4002 bp (g), respectively. Confirmation of the deletion of *Sc*.*ARO80* in IMK473 and *Sb*.*ARO80* in IMK474 by PCR-generated fragments of 2992 bp (f) and 2895 bp (h), respectively. The products obtained were then subjected to agarose gel electrophoresis.

The first condition to use *amdSYM* as selectable marker is that the parental strain does not have the capability to use acetamide as sole nitrogen source. To verify whether other laboratory strains besides the strains of the CEN.PK lineage could be modified using *amdSYM* as marker, the ability to grow on media containing acetamide as nitrogen source of three popular laboratory strains, namely CBS8066, YSBN [i.e. a prototrophic BY strain (Canelas *et al*., 2010)], and S288c (Mortimer & Johnston, 1986), was tested. None of the laboratory strains were able to use acetamide as sole nitrogen source (Fig. 5). To further expand the range of species in which *amdSYM* could be used, two brewing strains, the *S. cerevisiae* Scottish Ale strain and the *S. pastorianus* lager brewing strain CBS1483, and the wild yeast *S. eubayanus* CBS12357 were tested for their ability to grow on acetamide as sole nitrogen source. Similarly to the laboratory strains, none of these *Saccharomyces* species grew with acetamide as sole nitrogen source (Fig. 6A). The absence of endogenous acetamidase activity demonstrated that *amdSYM* could potentially be used as selectable marker in a wide range of laboratory, wild, and industrial *Saccharomyces* strains.

To confirm the universality of *amdSYM* as selectable marker, genes were deleted using *amdSYM* in the above-mentioned industrial and wild yeast strains. To compensate for the expected lower efficiency of homologous recombination in these non-*cerevisiae* species, the deletion cassettes were designed with longer homologous flanking regions of at least 500 bp. The gene *HXK1* was deleted in both the laboratory strain CEN.PK113-7D and the *S. pastorianus* lager brewing strain CBS1483 using cassettes containing *amdSYM*. *Saccharomyces pastorianus* is a hybrid species that contains two subgenomes: one that resembles *S. cerevisiae* genome and another one similar to *S. eubayanus* (Libkind *et al*., 2011). In this study, only the *S. cerevisiae* allele (*Sc.HXK1*) was deleted. The strains generated were named IMK468 (*Sc.hxk1Δ::amdSYM*) for the CEN.PK mutant and IMK470 (*Sc.hxk1Δ::amdSYM*) for the *S. pastorianus* mutant. Additionally, the *S. cerevisiae* Scottish Ale brewing strain and the recently described wild yeast *S. eubayanus* CBS12357 were also genetically modified using *amdSYM*. Making use of *amdSYM* as marker cassette the gene *Sb.ARO80* was deleted in *S. eubayanus* resulting in the strain IMK474, and *Sc.ARO80* was deleted in the *S. cerevisiae* Scottish Ale strain, generating the strain IMK473.

IMK468, IMK470, IMK473, and IMK474 all demonstrated the integration of *amdSYM* in their genomes by their ability to grow on acetamide as sole nitrogen source (Fig. 6A). PCR analysis confirmed the deletion of the targeted genes (Fig. 6B). The growth on acetamide plates was slower for industrial strains as compared to CEN.PK-derived deletion mutants, but increasing the acetamide concentration resulted in faster growth, thereby accelerating the screening process (data not shown). Therefore, the amount of acetamide necessary for *amdSYM*-based strain construction may be strain-dependent and requires optimization.

## Conclusions

When transformed into yeast, *A. nidulans amdS* conferred the capability to use acetamide as sole nitrogen source. This gain of function allowed the use of *amdS* as a new dominant marker in *S. cerevisiae*. In the present work, a new heterologous module, *amdSYM*, which encompasses the regulatory regions of *A. gossypii TEF2* and the *A. nidulans* gene *amdS*, was constructed and has been made available for the research community in the widely used plasmid series as pUG-amdSYM. Not only is *amdSYM* a new dominant marker, but it is also an additional counter-selectable marker in the yeast genetic toolbox. A strong feature of *amdSYM* is that, contrary to all other counter-selectable markers available, it does not require a specific genetic background for the strain to be selected.

Furthermore, while most marker recycling methods, such as the *Lox*P-*Cre* recombinase system, have the disadvantage of leaving scars after each recycling, in the present study, marker removal was scarless (Akada *et al*., 2006), leaving endogenous sequences after marker excision. In principle, *amdSYM* can be re-used an unlimited number of times, thus enabling multiple modifications without the protein burden that would cause the overexpression of several heterologous markers.

In conclusion, the new marker module *amdSYM* is an excellent tool for consecutive genetic modifications in the yeast *S. cerevisiae* and a good alternative to *URA3*, *MET15*, or *LYS2* with the additional substantial advantage that it is not limited to specific strain backgrounds or to the *cerevisiae* species. *amdSYM* has indeed been successfully used as selection marker to perform deletions in the newly discovered wild species of *S. eubayanus* and in *S. cerevisiae* and *S. pastorianus* brewing strains. Thanks to this later success in deleting genes from the aneuploid and hybrid genome of *S. pastorianus, amdS*YM is expected to considerably contribute to the functional analysis of genes in strains with complex genome architecture. As the first dominant recyclable marker, *amdSYM* opens the door to fast and easy genetic manipulation in *Saccharomyces* laboratory, wild, and industrial strains.
